# Efficacy and Tolerability of Two Quadruple Regimens: Bismuth, Omeprazole, Metronidazole with Amoxicillin or Tetracycline as First-Line Treatment for Eradication of *Helicobacter Pylori* in Patients with Duodenal Ulcer: A Randomized Clinical Trial

**DOI:** 10.1371/journal.pone.0197096

**Published:** 2018-06-11

**Authors:** Hassan Salmanroghani, Massoud Mirvakili, Mahmud Baghbanian, Roham Salmanroghani, Golshid Sanati, Pouria Yazdian

**Affiliations:** 1 Department of Internal Medicine, ShahidSadoughi University of Medical Sciences, Yazd, Iran; 2 Immunology Research Center, Tehran University of Medical Sciences, Tehran, Iran; University Hospital Llandough, UNITED KINGDOM

## Abstract

**Aim:**

To evaluate the efficacy and tolerability of tetracycline vs. high-dose amoxicillin in bismuth-based quadruple therapy for *Helicobacter pylori*(*H*. *pylori*) eradication.

**Methods:**

This randomized, open-label clinical trial included 228 patients with *H*.*pylori* infection and duodenal ulcer without a history of *H*.*pylori* treatment. Patients were randomly divided into two groups. The amoxicillin group received metronidazole 500mg, bismuth subcitrate 240mg, and amoxicillin 1000mg, all three times a day, plus omeprazole 20 mg twice a day, for 14 days. The tetracycline group received metronidazole 500mg three times a day; bismuth subcitrate240mg and tetracycline HCl 500mg, both four times a day; and omeprazole 20 mg twice a day, for 14 days. Evaluation for compliance and drug-relatedadverse effects were evaluated at the end of two weeks. Eight weeks after the end of treatment, the rate of *H*.*pylori* eradication was assessed by the C^13^urease breath test.

**Results:**

There were no significant demographic differences between the two groups. Eradication rate was higher with the amoxicillin-containing regimen than the tetracycline-containing regimen: 105/110 (95.51%; 95% confidence interval, 91.5%–99.3%) vs. 88/105 (83.8%; 95%CI, 76.7%–90.8%) by per-protocol analysis (*p* = 0.005) and 92.9% (95%CI, 88.1%–97.6%) vs. 76.5% (95%CI, 68.7%–84.2%) by intention-to-treat analysis (ITT, *p* = 0.001). Adverse effects were significant higher in the tetracycline groupthan in the amoxicillin group (65.2% vs. 43.4%; *p* = 0.001).

**Conclusion:**

Bismuth-based quadruple therapy including high-dose amoxicillin and metronidazole achieved an acceptable rate of *H*.*pylori* infection eradication with good tolerance in patients with duodenal ulcer. This regimen can overcome treatment resistance in areas with high prevalence of metronidazole and clarithromycin resistance.

**Trial registration:**

The Thai Clinical Trial Registry (TCTR) 20170623004

## Introduction

*Helicobacter pylori*(*H*. *pylori*) is a gram-negative bacterium that infects approximately 50% of people in industrialized nations and up to 80% in less-developed countries[1]. *H*. *pylori*causes peptic ulcer disease, chronic gastritis, gastric adenocarcinoma, and mucosa-associated lymphoid tissue lymphoma[2]. Although the bacterium is susceptible to most antimicrobial agents*invitro*, successful treatment of *H*.*pylori*remains a challenge[3].Factors contributing to success in*H*. *pylori* treatment are drug efficacy, host compliance, and bacterial resistance[1, 2]. Resistance to commonly used drugs such as metronidazole and clarithromycin, is the most important reason for treatment failure of current regimens[1]. Emerging evidence shows high resistance to clarithromycin in countries with high consumption of macrolidederivatives[4], whereas regimen containing clarithromycin was reported to achieve 80% eradication rate[5]. Previous studies from our region (Iran)revealed that more than 20% of *H*. *pylori* isolates were resistant to clarithromycin and that over 50% of the *H*. *pylori* isolates were resistant to metronidazole [6–8].

Due to high resistance to metronidazole and clarithromycin, regimens containing these antibiotics are not efficient[9]. In some studies on patients with metronidazole- and clarithromycin-resistant*H*. *pylori*, bismuth-based quadruple therapy was reported as a preferable regimen for eradication of *H*. *pylori*[10, 11]. This bismuth-based quadruple therapy includesbismuth, a proton pump inhibitor (PPI), and tetracycline together with metronidazole or tinidazole, with proper doses and duration. Quadruple regimen with suboptimal metronidazole doses (<1500 mg/day) was reported to achieve an overall eradication rate of 70% [3,4]. Increasing the dose of metronidazole in bismuth-based quadruple therapy was the first step in increasing eradication rates to acceptable levels, and bismuth compound was reported to be necessary for such response [12, 13]. However, low compliance,as well as increased side effects, are major issues that arise if full-dose metronidazole and tetracycline are used. There is also some evidence suggesting an increase in *H*.*pylori* resistance to tetracycline [14–16].

Outcomes with substitution of tetracycline with amoxicillin in bismuth-based quadruple therapy have not been widely studied, especially in countries with high *H*.*pylori* resistance to metronidazole and clarithromycin such as Iran. Our pilot study revealed that a very good *H*.*pylori* eradication rate could be achieved with a modified bismuth-based quadruple therapy containing high-dose amoxicillin (3 g/day), adequate-dose metronidazole (1.5 g/day), and a PPI.Therefore, this open-label, randomized clinical trial was designed to compare the classic bismuth-based quadruple regimen containing metronidazole (1500mg/day) with a modified bismuth-based quadruple therapy containing high-dose amoxicillin (3 g/day), metronidazole (1.5 g/day),and a PPI, with the aim to compare eradication rates, adverse effects, and patient compliance.

## Materials and methods

### Patient population

This was a prospective, randomized, open-label clinical trial study conducted at ShahidSadoughi University (SSU) of Medical Center, a tertiary care hospital located in Yazd, Iran. Criteria for inclusion in this study were as follows: no history of treatment for *H*.*pylori* eradication, age above 18 years, endoscopically confirmed diagnosis of duodenal ulcer, and positive rapidureasetest. Patients with a history of previous gastric surgery, allergy to antibiotics, those who were treated with antibiotics in the preceding eight weeks,those with major systemic disease, and those who were pregnantor lactating were excluded from the study. The primary endpoint was *H*.*pylori* eradication ratebyintention-to-treat (ITT) analysis. The secondary endpoint was frequency of adverse effects and treatment compliance.

### Registration

Ethics approval for this study was obtained from Yazd University Medical Sciences ethics committee on September 20, 2014, prior to patient enrollment. The clinical trial registration was completed retrospectively several months after patient enrollment was started, due to a miscommunication with the institution. We emphasize that our study begins and progress with complete consideration of ethicalrules. The authors confirm that all ongoing and related trials related to this study are registered.

### Intervention

Patients were enrolled between October 20, 2014 and July 15, 2016. After the patients provided consent to participate in this trial, they were randomly assigned at a 1:1 ratio to receive one of the following two treatment regimens. The amoxicillin group (group I) received metronidazole 500mg, amoxicillin 1000mg, bismuth subcitrate 240mg, all three times a day, plus omeprazole 20mg twice a day, for 14 days. The tetracycline group (group II) received omeprazole 20mg twice a day; bismuth 240 mg, and tetracycline HCl500 mg, both four times a day;and metronidazole 500 mg three times a day, for 14 days. Omeprazole and bismuth were taken before meals, and antibiotics were used after meals. All patients were instructed on potential adverse effects and kept under observation during treatment for evaluation of adverse effects and compliance. All patients were requested to record any adverse effects that occurred during therapy, including bad taste, diarrhea, dizziness, weakness, nausea, loss of appetite, vomiting, fatigue, fever, and skin rash. Severe adverse effects were defined as those that would be considered to disrupt daily activities that required treatment discontinuation by the patient.

### Outcomes

For evaluation for the primary outcome of *H*. *pylori* eradication rate, patients were asked to stop the PPI or the H2 blocker for at least four weeks before follow-up evaluation. Eight weeks after conclusion of the two-week study treatment, patients were assessed by the C^13^urease breath test by personnel who were blinded to the treatment, and a value of less than 4% was defined as successful*H*. *pylori* eradication. For evaluation of secondary outcomes, data on adverse effects were collected through a standard sideeffect questionnaire, and good compliance was defined as ingesting more than 80% of the total number of doses included in the regimen.

### Sampling and blinding

Sample size for the trial was calculated based on the following assumptions. Average rate of successfully achieved eradication of *H*.*pylori*with the standard quadruple therapy was 80% [17]. Based onourpilot study results showing an *H*.*pylori* eradication rate of 94% with the modified bismuth-based quadruple therapy containing high-dose amoxicillin (3 g/day),adequate-dose metronidazole dose (1.5 g/day) and omeprazole, we chose a two-sided alpha value of 0.05, and a power of 80%.Based on these assumptions at least 208 participates (104 subjects in each groups) would be required. In order to accommodate a 10% rate of lost to follow-up, we enrolled 228 patients.

### Statistical methods

All registered data were analyzed using SPSS software version 22 for Windows (SPSS, Chicago, IL). Data were presented as means with standard deviation (SD), frequencies, and percentages. The chi-square and Fisher’s exact tests were used for comparison of categorical data between the two groups. *P* values of less than 0.05 were considered significant for all analyses. ITT and per-protocol (PP)analyses were performed to calculate eradication rates. The ITT analysis included all randomized patients. Individuals who did not take at least 80% of the drugs andthosewith unknown post-treatment *H*. *pylori* status were excluded from the PP analysis. Odds ratios with 95% confidence intervals (CIs) were calculated where appropriate.

### Ethical consideration

This study was approved by the Ethics Committee of ShahidSadoughiUniversityof Medical Sciences in Yazd, Iran and registered with the protocol number ˮIr.ssu.rec.1394.13712”on September 20, 2014. Participants provided written informed consent and were included in the study after they were provided information on treatment methods. This trial was also registered with Thai Clinical Trial Registry (Number: TCTR20170623004). The Consort 2010 checklist, the study protocol and the Ethic approval of study are given S1 Checklist, S1 File and S2 File.

http://dx.doi.org/10.17504/protocols.io.mdjc24n

## Results

### Patient characteristics and compliance

**Fig 1:**
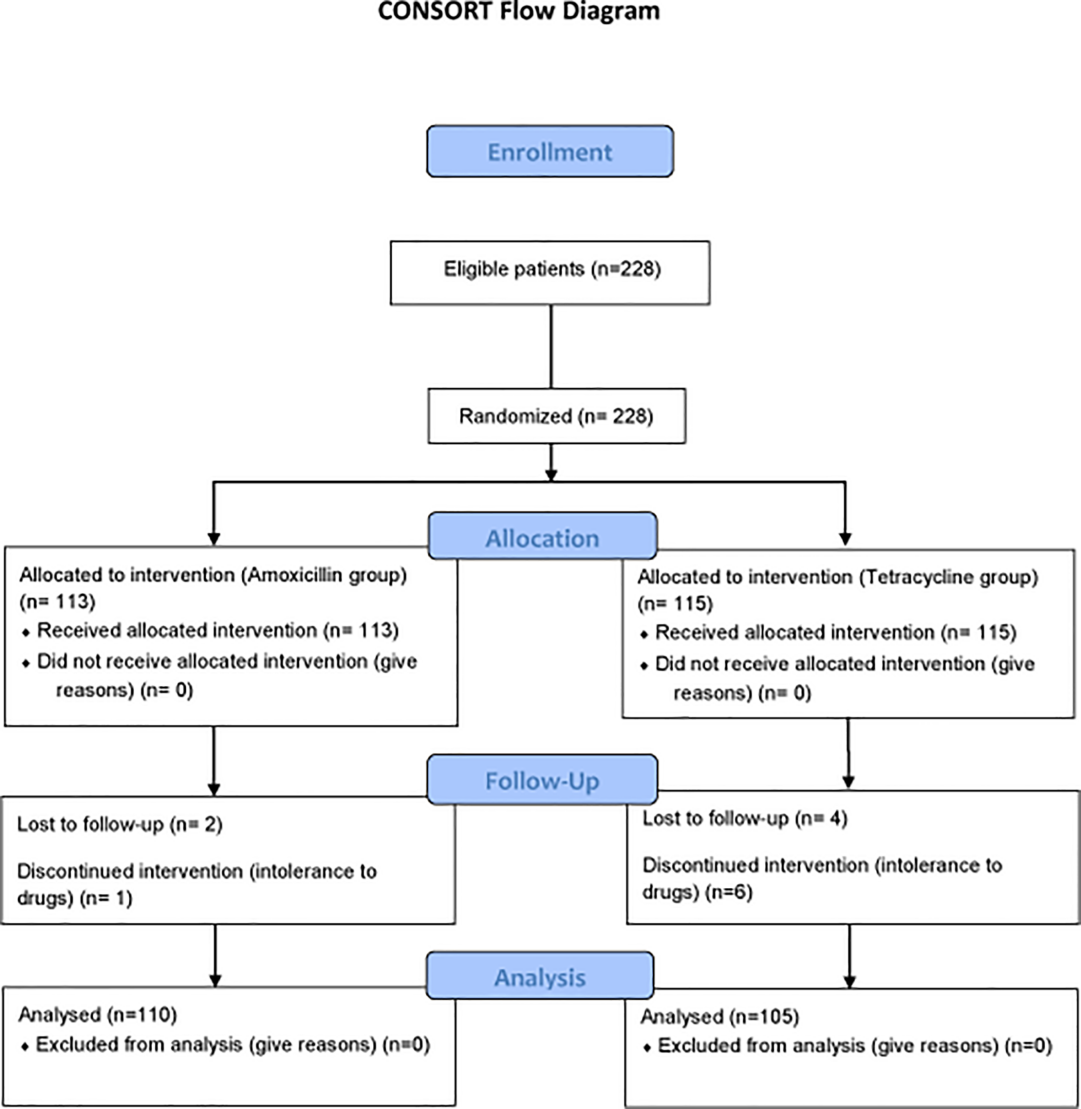
CONSORT flow diagram.

A total of 228 patients who were eligible were recruited from the SSU Gastroenterology Department. There were no significant differences in baseline characteristics including age and sex between the two treatment groups (Table 1). All patients except those who were lost to follow-up and those who could not tolerate the regimen were assessed by the C^13^urease breath test after the conclusion of treatments. The CONSORTflow diagram is depicted in Fig 1.

**Table 1:** Baseline characteristics of patients randomized in the two groups.

Variables		Amoxicillin group	Tetracycline group	P value
Number of subjects		113	115	
Age (mean±SD) and range		41.8±13.96 (19–72)	39.84±14.36 (18–74)	0.29*
Gender	Male	61	56	0.42**
	Female	52	59	
Lost to follow up		2	4	
Cannot tolerate drugs		1	6	
Compliance (taken 80% of tablets)		(110/113)98.23%	(105/115)91.30%	

* Independent Sample T Test;

**Pearson Chi Square Test

### H. pylori eradication rates

Overall, the eradication rate by per-protocol analysis was 105/110 (95.51%; 95%CI, 91.5%–99.3%) in group I and 88/105 (83.8%; 95%CI, 76.7%–90.8%) in group II, which was significantly different between the two treatment groups (*p* = 0.005). As presented in Table 2, the ITT analysis also revealed that the eradication rates were significantly different between the amoxicillin and tetracycline groups (92.9%; 95%CI, 88.1%–97.6% and 76.5%; 95%CI, 68.7%–84.2%, respectively; *p*<0.001). These results clearly showed that the *H*. *pylori* eradication rate was significantly higher in the amoxicillin group than in the tetracycline group.

**Table 2:** H.pylorieradication rates in intention-to-treat and per-protocol analyses.

Analysis	Amoxicillin group	Tetracycline group	95% CI for difference between amoxicillin and tetracycline group	Mean difference	p value
ITT	92.9% (105/113)	76.5% (88/115)	7.3% to 25.4%	16.3%	0.001*
95% CI	88.1% to 97.6%	68.7% to 84.2%			
PP	95.5% (105/110)	83.8% (88/105)	3.5% to 19.6%	11.6%	0.005*
95% CI	91.5% to 99.3%	76.7% to 90.8%			

*Pearson Chi Square;

**ITT**: intention-to-treat; **PP**: per protocol; **CI**: confidence interval

### Adverse effects and compliance

Overall, 4 patients in the tetracycline group and 2 patients in the amoxicillin group were lost to follow-up, and no outcome data were available for these patients. Additionally, 6 patients in the tetracycline group and 1 patient in the amoxicillin group could not tolerate the medication, i.e.,failed to take at least 80% of the prescribed medication, due to severe adverse effects that started a few days after initiation of the treatment. Adverse effects included tolerable diarrhea, bad taste, dizziness, weakness, nausea, skin rash, fever, fatigue, and vomiting, which were reported by 43.4% (49 of 113) of the patients in the amoxicillin group and 65.2% (75 of 115) of the patients in the tetracycline group, and adverse effects were significantly more frequent in the tetracycline group (*p* = 0.001). Bad taste was the most common side effect in both groups, whereas the frequency of vomiting was significantly higher in the tetracycline treatment group (4 vs. 0 in tetracycline vs.amoxicillin groups; *p* = 0.04).A summary of adverse effects in this study is presented in Table 3.

**Table 3:** Side effects of treatment in groups.

Variables	Amoxicillin group	Tetracycline group	P Value*
Tolerable diarrhea	6 (5.3%)	8 (7.0%)	0.60
Bad taste	19 (16.8%)	22 (19.1%)	0.64
Dizziness	3 (2.7%)	4 (3.5%)	0.71
Weakness	4 (3.5%)	6 (5.2%)	0.61
Nausea	9 (8.0%)	17 (14.8%)	0.10
Skin rash	4 (3.5%)	4(3.5%)	0.98
Fever	2 (1.8%)	3 (2.6%)	0.66
Fatigue	2 (1.8%)	7 (6.1%)	0.09
Vomiting	0 (0%)	4 (3.5%)	0.04
Total side effects % (n)	49 (43.4%)	75 (65.2%)	0.001

*Pearson Chi Square Test

## Discussion

Results of this open-label randomized study revealed that substitution of tetracycline with high-dose amoxicillin in classic bismuth-based quadruple therapy can increase *H*. *pylori* eradication rate and compliance in patients with duodenal ulcer. In patients who received high-dose amoxicillin in bismuth-based quadruple therapy, the eradication rates were 95.6% and 92.9% by the PP and ITT analyses, respectively. Conversely, the eradication rates in patients who received classic bismuth-based quadruple therapy were 83.3% and 75% by PP and ITT analyses, respectively. These findings indicatedthat substitution of tetracycline with high-dose amoxicillin significantly increased the eradication rate not only by the ITT analysis but also the PP analysis.As more than 50% of *H*. *pylori* strains in Iran are resistant to metronidazole[6–8], amoxicillin might achieve better synergistic effects with metronidazole than tetracycline for eradication of metronidazole-resistant*H*. *pylori* strains. Additionally, patients had better tolerance to bismuth-based quadruple therapy containing amoxicillin instead of tetracycline.

Generally, management of disorders associated with *H*. *pylori* infection requires treatment regimens that have eradication rates of more than 90% to 95%, if possible [18]. Use of similar effective first-line treatments is important not only for disease cure but also for prevention of secondary antibiotic resistance [19].

A recent review of various regimens used as primary treatment in West Asia suggested that bismuth-furazolidone-metronidazole treatment for 10 days, clarithromycin-based hybrid treatment for 14 days, and classic bismuth-based quadruple therapy including PPIs, bismuth, tetracycline, and metronidazole for 14 days,wereequally effective [20]. Since the furazolidone-based regimen has many side effects, the regimen containing adequate-dose furazolidone (400mg/day) is not recommended [21, 22]. Due to high resistance to clarithromycin, clarithromycin-containing regimen isnot acceptable in our treatment course [23]. Potentially high resistance to levofloxacin also limits its use as front-line treatment [9, 23]. *H*. *pylori* culture and antibiogram are expensive, and lack of information on pretreatment susceptibility to antibiotics is the main reason for bismuth-based quadruple therapy as a primary regimen for eradication of *H*. *pylori* in our region, as recommended by the Toronto Consensus [24]. Malfertheiner and colleagues showed that in regions with high resistance to clarithromycin, quadruple treatment containing bismuth, metronidazole, tetracycline, and a PPI was preferable as the primary therapy for *H*. *pylori* eradication. Quadruple therapy achieves superior eradication with safety and tolerability comparable to those obtained with standard triple therapy [25]. In contrast to metronidazole, increase in clarithromycin dose was reported not to increase the eradication rate of clarithromycin-resistant *H*. *pylori*[26].

Liang *et al*. concluded that four different bismuth-based quadruple treatments exhibited eradication ratesof>90% against *H*. *pylori* in subjects who were unresponsive to prior therapies, including those showing resistance to clarithromycin and metronidazole [27].Therefore, the two-week bismuth-based quadruple therapy containing high-dose metronidazole, a PPI, and tetracycline is a proper first-line treatment regimen in our patients with duodenal ulcer [28–30]. This regimen requires modifications with respect to drugs, doses, and duration to reach excellent *H*. *pylori* eradication rates. Bismuth and metronidazole are necessaryadditionsin this regimen. Increase in *H*. *pylori*eradication rate with an increased metronidazole dose is dependent on the presence of bismuth. Bismuth also improves the elimination of*H*. *pylori* by triple therapy regimen [31]. In a systematic review and meta-analysis of 35 randomized clinical trials and 4763 subjects, the safety profile of bismuth was assessed, which revealed insignificant side effects in patients undergoing bismuth-based treatment [32].Emerging evidence of increasing resistance to tetracycline and reports demonstrating that adequate-dose metronidazole in combination with tetracycline results in more side effects raise concerns regarding substitution of tetracycline with another antibiotic.

To achieve increased compliance to classic bismuth-based quadruple therapy, tetracycline was replaced with high-dose amoxicillin, based on several lines of evidence:In a recent study, high-dose amoxicillin (750mg four times a day) in combination with a potent PPI achieved a very good eradication rate, suggesting that amoxicillin is a unique anti-*H*. *pylori* antibiotic that has an acceptable eradication rate when used as part of a dual therapy containing a PPI [33, 34].The resistance rates to amoxicillin, metronidazole, and clarithromycin were about 2%, 44%, and 29% in America; 0.7%, 35%, and 18% in Europe; and 2%, 38%, and 21% in Asia, respectively [35]. *H*. *pylori*was found to rapidly acquire antibiotic resistance to clarithromycin, metronidazole, and levofloxacin but not to amoxicillin after a single course of anti-*H*. *pylori* therapy [36]. One potential explanation for the difference in acquired antibiotic resistance is that a single-point mutation can lead to resistance to clarithromycin, metronidazole, and levofloxacin, whereas multiple-site mutations are necessary to confer amoxicillin resistance [37–39]. A recent study among US male patients revealed increased resistance to clarithromycin and levofloxacin but no resistance to amoxicillin [40]. In Iran, 1.6%, 16.7%, and 57.5% of*H*. *pylori* strainsshowed resistance to amoxicillin, clarithromycin, and metronidazole, respectively; however, no tetracycline resistance was reported [41]. However, this sensitivity pattern was found to have changed five years later, and there is an increasing resistance to tetracycline [42]. A recent study in Yazd, Iran revealed that *H*.*pylori* is more resistant to tetracycline than amoxicillin[43]. In a study in China, amoxicillin, in combination with metronidazole, bismuth, and lansoprazole, eradicated metronidazole-resistant*H*. *pylori*. In their study, Zhang *et al*. used 2g amoxicillin per day in combination with lansoprazole, metronidazole, and bismuth [44]. Lansoprazole, albeit more potent, is more expensive than omeprazole in Iran; therefore, high-dose amoxicillin with omeprazole was preferred over 2g amoxicillin with lansoprazole.

The ITT analysis revealed an eradication rate of 92.92% in the amoxicillin group compared with the rate of 76.5% in the tetracycline group, indicating that bismuth-based quadruple therapy containing tetracycline with adequate-dose metronidazole was associated with more adverse effects and intolerance. Unfortunately, severe adverse effects that lead to intolerance in the tetracycline group occurred 4–5 days after treatment initiation. In contrast, there were no severe drug-associated complications with the high-dose amoxicillin regimen, and the patient compliance was good.

Inability to evaluate the *H*. *pylori*antimicrobial resistance patterns was one of the main limitations of this study; howevera recent regional study indicated that*H*. *pylori* resistance to tetracycline was more common than that to amoxicillin, which might explain the 16% failure rate in the tetracycline groupcompared to the 5% failure rate in the amoxicillin group based on the PP analysis[43].

## Conclusion

The results of this randomized, open-label clinical trial revealed that a two-week bismuth-based quadruple therapy including high-dose amoxicillin and metronidazole had an acceptable eradication rate with good tolerance in patients with duodenal ulcer and *H*. *pylori* infection. This therapy might overcome treatment resistance in areas with high prevalence of *H*. *pylori* resistance to metronidazole and clarithromycin.

## Supporting information

S1 Checklist CONSORT 2010 checklist. (DOC)

S1 File Study protocol. (DOC)

S2 File Ethic approval. (PDF)

## References

[1] Go M. Natural history and epidemiology of Helicobacter pylori infection. Alimentary pharmacology & therapeutics. 2002;16(s1):3–15. 10.1046/j.1365-2036.2002.0160s1003.x 11849122

[2] Khademi F, Poursina F, Hosseini E, Akbari M, Safaei HG. Helicobacter pylori in Iran: A systematic review on the antibiotic resistance. Iranian journal of basic medical sciences. 2015;18(1):2 25810869 PMC4366738

[3] Fischbach L, Evans E. Meta‐analysis: the effect of antibiotic resistance status on the efficacy of triple and quadruple first‐line therapies for Helicobacter pylori. Alimentary pharmacology & therapeutics. 2007;26(3):343–57. 17635369 10.1111/j.1365-2036.2007.03386.x

[4] Lu H, Zhang W, Graham DY. Bismuth-containing quadruple therapy for Helicobacter pylori: lessons from China. European journal of gastroenterology & hepatology. 2013;25(10). 10.1097/MEG.0b013e3283633b57 PMC3865448 23778309

[5] Graham DY, Fischbach L. Helicobacter pylori treatment in the era of increasing antibiotic resistance. Gut. 2010;59(8):1143–53. doi: 10.1136/gut.2009.192757 20525969 10.1136/gut.2009.192757

[6] Shokrzadeh L, Alebouyeh M, Mirzaei T, Farzi N, Zali MR. Prevalence of multiple drug-resistant Helicobacter pylori strains among patients with different gastric disorders in Iran. Microbial Drug Resistance. 2015;21(1):105–10. doi: 10.1089/mdr.2014.0081 25303151 10.1089/mdr.2014.0081

[7] KeshavarzAziziRaftar S, Moniri R, Saffari M, RazaviZadeh M, Arj A, Mousavi SGA, et al The helicobacter pylori resistance rate to clarithromycin in Iran. Microbial Drug Resistance. 2015;21(1):69–73. doi: 10.1089/mdr.2014.0104 25144338 10.1089/mdr.2014.0104

[8] Farshad S, Alborzi A, Japoni A, Ranjbar R, Asl KH, Badiee P, et al Antimicrobial susceptibility of Helicobacter pylori strains isolated from patients in Shiraz, Southern Iran. World Journal of Gastroenterology: WJG. 2010;16(45):5746 doi: 10.3748/wjg.v16.i45.5746 21128326 10.3748/wjg.v16.i45.5746 PMC2997992

[9] Graham DY, Lee YC, Wu MS. Rational Helicobacter pylori therapy: evidence-based medicine rather than medicine-based evidence. Clinical Gastroenterology and Hepatology. 2014;12(2):177–86. e3. doi: 10.1016/j.cgh.2013.05.028 23751282 10.1016/j.cgh.2013.05.028 PMC3830667

[10] Fischbach L, Zanten S, Dickason J. Meta‐analysis: the efficacy, adverse events, and adherence related to first‐line anti‐Helicobacter pylori quadruple therapies. Alimentary pharmacology & therapeutics. 2004;20(10):1071–82. 15569109 10.1111/j.1365-2036.2004.02248.x

[11] Calvet X, Ducons J, Guardiola J, Tito L, Andreu V, Bory F, et al One‐week triple vs. quadruple therapy for Helicobacter pylori infection—a randomized trial. Alimentary pharmacology & therapeutics. 2002;16(7):1261–7. 12144575 10.1046/j.1365-2036.2002.01278.x

[12] Goodwin C, Marshall B, Blincow E, Wilson D, Blackbourn S, Phillips M. Prevention of nitroimidazole resistance in Campylobacter pylori by coadministration of colloidal bismuth subcitrate: clinical and in vitro studies. Journal of clinical pathology. 1988;41(2):207–10. 3280609 10.1136/jcp.41.2.207 PMC1141380

[13] Laine L, Hunt R, El-Zimaity H, Nguyen B, Osato M, Spénard J. Bismuth-based quadruple therapy using a single capsule of bismuth biskalcitrate, metronidazole, and tetracycline given with omeprazole versus omeprazole, amoxicillin, and clarithromycin for eradication of Helicobacter pylori in duodenal ulcer patients: a prospective, randomized, multicenter, North American trial. The American journal of gastroenterology. 2003;98(3):562–7. 12650788 10.1111/j.1572-0241.2003.t01-1-07288.x

[14] Abadi AT, Taghvaei T, Mobarez AM, Carpenter BM, Merrell DS. Frequency of antibiotic resistance in Helicobacter pylori strains isolated from the northern population of Iran. Journal of microbiology (Seoul, Korea). 2011;49(6):987–93. 10.1007/s12275-011-1170-6 PMC3275342 22203563

[15] Kim JJ, Reddy R, Lee M, Kim JG, El-Zaatari FA, Osato MS, et al Analysis of metronidazole, clarithromycin and tetracycline resistance of Helicobacter pylori isolates from Korea. The Journal of antimicrobial chemotherapy. 2001;47(4):459–61. 11266421 10.1093/jac/47.4.459

[16] Mendonca S, Ecclissato C, Sartori MS, Godoy AP, Guerzoni RA, Degger M, et al Prevalence of Helicobacter pylori resistance to metronidazole, clarithromycin, amoxicillin, tetracycline, and furazolidone in Brazil. Helicobacter. 2000;5(2):79–83 10849055 10.1046/j.1523-5378.2000.00011.x

[17] A Fischbach L, Evans EL Meta-analysis: the effect of antibiotic resistance status on the efficacy of triple and quadruple first-line therapies for Helicobacter pylori. Aliment PharmacolTher. 2007 8 1; 26(3):343–57. 10.1111/j.1365-2036.2007.03386.x 17635369

[18] Graham DY. Efficient identification and evaluation of effective Helicobacter pylori therapies. Clinical Gastroenterology and Hepatology. 2009;7(2):145–8. doi: 10.1016/j.cgh.2008.10.024 19026766 10.1016/j.cgh.2008.10.024 PMC2838433

[19] Huang J, Hunt R. Treatment after failure: the problem of “non-responders”. Gut. 1999;45(suppl 1):I40–I4. 10457036 10.1136/gut.45.2008.i40 PMC1766657

[20] Fakheri H, Bari Z, Aarabi M, Malekzadeh R. Helicobacter pylori eradication in West Asia: a review. World Journal of Gastroenterology: WJG. 2014;20(30):10355 doi: 10.3748/wjg.v20.i30.10355 25132752 10.3748/wjg.v20.i30.10355 PMC4130843

[21] Roghani HS, Massarrat S, Shirekhoda M, Butorab Z. Effect of different doses of furazolidone with amoxicillin and omeprazole on eradication of Helicobacter pylori. Journal of gastroenterology and hepatology. 2003;18(7):778–82. 12795748 10.1046/j.1440-1746.2003.03058.x

[22] Sadeghifard N, Seidnazari T, Ghafourian S, Soleimani M, Maleki A, Qomi MA, et al Survey in Iran of clarithromycin resistance in Helicobacter pylori isolates by PCR-RFLP. Southeast Asian Journal of Tropical Medicine and Public Health. 2013;44(1):89 23682442

[23] De Francesco V, Giorgio F, Hassan C, Manes G, Vannella L, Panella C, et al Worldwide H. pylori antibiotic resistance: a systematic review. Journal of Gastrointestinal & Liver Diseases. 2010;19(4). 21188333

[24] Fallone CA, Chiba N, van Zanten SV, Fischbach L, Gisbert JP, Hunt RH, et al The Toronto consensus for the treatment of Helicobacter pylori infection in adults. Gastroenterology. 2016;151(1):51–69. e14. doi: 10.1053/j.gastro.2016.04.006 27102658 10.1053/j.gastro.2016.04.006

[25] Delchier J, Malfertheiner P, Thieroff‐Ekerdt R. Use of a combination formulation of bismuth, metronidazole and tetracycline with omeprazole as a rescue therapy for eradication of Helicobacter pylori. Alimentary pharmacology & therapeutics. 2014;40(2):171–7. 24863854 10.1111/apt.12808

[26] Murakami K, Sato R, Okimoto T, Nasu M, Fujioka T, Kodama M, et al Eradication rates of clarithromycin‐resistant Helicobacter pylori using either rabeprazole or lansoprazole plus amoxicillin and clarithromycin. Alimentary pharmacology & therapeutics. 2002;16(11):1933–8. 12390102 10.1046/j.1365-2036.2002.01368.x

[27] Liang X, Xu X, Zheng Q, Zhang W, Sun Q, Liu W, et al Efficacy of bismuth-containing quadruple therapies for clarithromycin-, metronidazole-, and fluoroquinolone-resistant Helicobacter pylori infections in a prospective study. Clinical Gastroenterology and Hepatology. 2013;11(7):802–7. e1. doi: 10.1016/j.cgh.2013.01.008 23376004 10.1016/j.cgh.2013.01.008

[28] Salazar CO, Cardenas VM, Reddy RK, Dominguez DC, Snyder LK, Graham DY. Greater than 95% success with 14‐day bismuth quadruple anti‐helicobacter pylori therapy: a pilot study in US Hispanics. Helicobacter. 2012;17(5):382–90. doi: 10.1111/j.1523-5378.2012.00962.x 22967122 10.1111/j.1523-5378.2012.00962.x

[29] Roghani HS, Massarrat S, Pahlewanzadeh M, Dashti M. Effect of two different doses of metronidazole and tetracycline in bismuth triple therapy on eradication of Helicobacter pylori and Its resistant strains. European journal of gastroenterology & hepatology. 1999;11(7):709–12. 10445787 10.1097/00042737-199907000-00004

[30] Graham D, Osato M, Hoffman J, Opekun A, Anderson S, Kwon D, et al Metronidazole containing quadruple therapy for infection with metronidazole resistant Helicobacter pylori: a prospective study. Alimentary Pharmacology and Therapeutics. 2000;14(6):745–50. 10848658 10.1046/j.1365-2036.2000.00770.x

[31] Dore MP, Lu H, Graham DY. Role of bismuth in improving Helicobacter pylori eradication with triple therapy. Gut. 2016;65(5):870–8. doi: 10.1136/gutjnl-2015-311019 26848181 10.1136/gutjnl-2015-311019

[32] Ford AC, Malfertheiner P, Giguère M, Santana J, Khan M, Moayyedi P. Adverse events with bismuth salts for Helicobacter pylori eradication: systematic review and meta-analysis. World journal of gastr10.3748/wjg.14.7361 PMC2778120 19109870

[33] Yang J-C, Lin C-J, Wang H-L, Chen J-D, Kao JY, Shun C-T, et al High-dose dual therapy is superior to standard first-line or rescue therapy for Helicobacter pylori infection. Clinical Gastroenterology and Hepatology. 2015;13(5):895–905. e5. doi: 10.1016/j.cgh.2014.10.036 25460556 10.1016/j.cgh.2014.10.036 PMC4404168

[34] Zullo A, Ridola L, De Francesco V, Gatta L, Hassan C, Alvaro D, et al High-dose esomeprazole and amoxicillin dual therapy for first-line Helicobacter pylori eradication: a proof of concept study. Annals of gastroenterology: quarterly publication of the Hellenic Society of Gastroenterology. 2015;28(4):448. PMC4585390 26423014

[35] Sun Q-J, Liang X, Zheng Q, Gu W-Q, Liu W-Z, Xiao S-D, et al Resistance of Helicobacter pylori to antibiotics from 2000 to 2009 in Shanghai. World journal of gastroenterology: WJG. 2010;16(40):5118 doi: 10.3748/wjg.v16.i40.5118 20976850 10.3748/wjg.v16.i40.5118 PMC2965290

[36] Berry V, Jennings K, Woodnutt G. Bactericidal and morphological effects of amoxicillin on Helicobacter pylori. Antimicrobial agents and chemotherapy. 1995;39(8):1859–61. 7486933 10.1128/aac.39.8.1859 PMC162840

[37] Rimbara E, Noguchi N, Kawai T, Sasatsu M. Mutations in penicillin-binding proteins 1, 2 and 3 are responsible for amoxicillin resistance in Helicobacter pylori. Journal of antimicrobial chemotherapy. 2008;61(5):995–8. doi: 10.1093/jac/dkn051 18276599 10.1093/jac/dkn051

[38] Yang J-C, Lu C-W, Lin C-J. Treatment of Helicobacter pylori infection: current status and future concepts. World Journal of Gastroenterology: WJG. 2014;20(18):5283 doi: 10.3748/wjg.v20.i18.5283 24833858 10.3748/wjg.v20.i18.5283 PMC4017043

[39] Megraud F. Helicobacter pylori and antibiotic resistance. Gut. 2007;56(11):1502–. doi: 10.1136/gut.2007.132514 17938430 10.1136/gut.2007.132514 PMC2095668

[40] Shiota S, Reddy R, Alsarraj A, El-Serag HB, Graham DY. Antibiotic resistance of Helicobacter pylori among male United States veterans. Clinical Gastroenterology and Hepatology. 2015;13(9):1616–24. doi: 10.1016/j.cgh.2015.02.005 25681693 10.1016/j.cgh.2015.02.005 PMC6905083

[41] Mohammadi M, Doroud D, Mohajerani N, Massarrat S. Helicobacter pylori antibiotic resistance in Iran. World Journal of Gastroenterology: WJG. 2005;11(38):6009 doi: 10.3748/wjg.v11.i38.6009 16273615 10.3748/wjg.v11.i38.6009 PMC4436725

[42] Massarrat S, Sheykholeslami A. Increase in resistance rates of H. pylori isolates to metronidazole and tetracycline-comparison of three 3-year studies. Archives of Iranian medicine. 2010;13(3):177 20433221

[43] Navidifar T, Eslami, Akhondi M, Baghbanian M, Zadeh HF, Zandi H. Antibacterial resistance patterns of helicobacter pyloriclinical isolates from gastric biopsy of patients in yazd. Int J Enteric Pathog. 2014;2(2):e17791.

[44] Zhang W, Chen Q, Liang X, Liu W, Xiao S, Graham DY, et al Bismuth, lansoprazole, amoxicillin and metronidazole or clarithromycin as first-line Helicobacter pylori therapy. Gut. 2015:gutjnl-2015-30990 10.1136/gutjnl-2015-309900 26338726

