# Supplementary material for: Efficacy and Tolerability of Two Quadruple Regimens: Bismuth, Omeprazole, Metronidazole with Amoxicillin or Tetracycline as First-Line Treatment for Eradication of Helicobacter Pylori in Patients with Duodenal Ulcer: A Randomized Clinical Trial
Source: PLoS One. 2018 Jun 11;13(6):e0197096. doi: 10.1371/journal.pone.0197096 (PMC5995362; doi:10.1371/journal.pone.0197096)
Supplement: S1 File — (DOC) [file pone.0197096.s002.doc]

**Study protocol**

**Efficacy and Tolerability of Two Quadruple Regimens: Bismuth, Omeprazole, Metronidazole with Amoxicillin or Tetracycline as First-Line Treatment for Eradication of *Helicobacter Pylori* in Patients with Duodenal Ulcer: A Randomized Clinical Trial**

**Correspondence Author:**

Hassan Salmanroghani, MD

Protocol summary

| Prospective, randomized, open label clinical trial | **Study design** |
| --- | --- |
| The objective is to evaluate the efficacy and tolerability of two quadruple regimens, Metronidazole 500mg , Bismuth subcitrate240mg , and Amoxicillin 1000mg and omeprazole 20mg (amoxicillin group) versus, bismuth 240 mg, tetracycline HCl 500 mg, metronidazole 500 mg and omprazole 20mg (tetracycline group) as first line treatment for Helicobacter pylori eradication | **Objective** |
| H. pylori infection Resistance to effective antibiotic such as clarithromycin and metronidazole is the most important reason for treatment failure. Pretreatment antibiotic sensitivity test is not simple and not recommended at present time. Recent expert guide line recommendation is use of Classic Bismuth- based quadruple therapy regimen as the first line treatment for eradication of H. pylori in area with High H. pylori resistance to metronidazole and clarithromycin. But there are three problem with this regimen:  1: Low compliance; because concomitant use of adequate-dose of metronidazole (1500 mg/day) with full dose of tetracycline (500 mg four times a day) are associated with frequent adverse event.  2: Emerging evidence suggest of increasing H. pylori resistance to tetracycline  3: No access to tetracycline in some countries.  Substitution of tetracycline with high dose of amoxicillin can solve these problems. | **Study hypotheses** |
| 1. Amoxicillin group: Metronidazole 500mg , Amoxicillin 1000mg , Bismuth subcitrate 240mg ,all three times a day and omeprazole 20mg twice a day for Two weeks  2.Tetracycline group: metronidazole 500 mg three times a day, bismuth 240 mg, tetracycline HCl 500 mg, both four times a day, and omprazole 20mg twice a day for Two weeks. | **Regimens** |
| 228 patients with duodenal ulcer and positive rapid urease test with no history of previous treatment for H. pylori Infection were enrolled from Gastroenterology clinic, Shahid sadoughi Medical Center, in Yazd, Iran. | **Number of subjects** |
| The primary end points of the study are:  Comparing the eradication rate between two bismuth-based quadruple regimens. H. pylori eradication is defined as a negative C-urea breath test beginning 8 weeks after the end of study | **Primary endpoints and measurement** |
| Study follow-up period was 10 weeks. | **Study follow up** |
| The secondary end points:  Adverse effects of these two quadruple treatment included (tolerable diarrhea, bad taste, dizziness, weakness, nausea, skin rash, fever, fatigue, and vomiting)  Rate of compliance to the prescribed therapy (more than 80% of medications used for all medications) | **Secondary endpoints** |
| Sample size for the trial was calculated based on the following assumptions. Average rate of successfully achieved eradication of *H. pylori* with the standard quadruple therapy was 80%. Based on our pilot study results showing an *H. pylori* eradication rate of 94% with the modified bismuth-based quadruple therapy containing high-dose amoxicillin (3 g/day), adequate-dose metronidazole dose (1.5 g/day) and omeprazole, we chose a two-sided alpha value of 0.05, and a power of 80%. Based on these assumptions at least 208 participate (104 subjects in each groups) would be required. In order to accommodate a 10% rate of lost to follow-up, we enrolled 228 patients. | **Sample size consideration** |
| 1.signed informed consent  2. no history of H. pylori infection eradication treatment  3.age above 18 years  4. Positive rapid urease test.  5.Duodenal ulcer | **inclusion criteria** |
| 1) Age under 18  2) History of gastric surgery  3) Gastric malignancy, including adenocarcinoma and lymphoma  4) Allergy reaction to antibiotics  5) Pregnant or lactating women  6) Treatment with antibiotics in the preceding eight weeks  7) Severe systemic disease or malignancy | **Exclusion criteria** |
| Salmanroghani hassan, MD , Gastroenterology Department, Shahid sadoughi Medical Center | **Principal study investigator** |
| Shahid sadoughi Clinical Research Center  Head: fallah zadah abarkoohi Hosein, MD | **Data management and analysis** |
| Shahid sadoughi Medical Center, Yazd, Iran | **Study site** |
| 10/2014  Enrollment period: 20 months  We used random allocation software for producing random allocation code | **Estimated start data and enrollment phase**  **Randomization:** |

**Introduction:**

*Helicobacter pylori* (*H. pylori*) are a gram-negative bacterium that infects approximately 50% of people in industrialized nations and up to 80% in less-developed countries (1). *H. pylori* cause peptic ulcer disease, chronic gastritis, gastric adenocarcinoma, and mucosa-associated lymphoid tissue lymphoma (2). Although the bacterium is susceptible to most antimicrobial agents *invitro*, successful treatment of *H. pylori* remains a challenge (3). Factors contributing to success in *H. pylori* treatment are drug efficacy, host compliance, and bacterial resistance (1, 2). Resistance to commonly used drugs such as metronidazole and clarithromycin is the most important reason for treatment failure of current regimens (1). Emerging evidence shows high resistance to clarithromycin in countries with high consumption of macrolide derivatives (4), whereas regimen containing clarithromycin was reported to achieve 80% eradication rate (5). Previous studies from our region (Iran) revealed that more than 20% of *H. pylori* isolates were resistant to clarithromycin and that over 50% of the *H. pylori* isolates were resistant to metronidazole (6-8).

Due to high resistance to metronidazole and clarithromycin, regimens containing these antibiotics are not efficient (9). In some studies on patients with metronidazole- and clarithromycin-resistant *H. pylori*, bismuth-based quadruple therapy was reported as a preferable regimen for eradication of *H. pylori* (10, 11). This bismuth-based quadruple therapy includes bismuth, a proton pump inhibitor (PPI), and tetracycline together with metronidazole or tinidazole, with proper doses and duration. Quadruple regimen with suboptimal metronidazole doses (<1500 mg/day) was reported to achieve an overall eradication rate of 70% (3, 4). Increasing the dose of metronidazole in bismuth-based quadruple therapy was the first step in increasing eradication rates to acceptable levels, and bismuth compound was reported to be necessary for such response (12, 13). However, low compliance, as well as increased side effects, is major issues that arise if full-dose metronidazole and tetracycline are used. There is also some evidence suggesting an increase in *H. pylori* resistance to tetracycline (14-16).

Outcomes with substitution of tetracycline with amoxicillin in bismuth-based quadruple therapy have not been widely studied, especially in countries with high *H. pylori* resistance to metronidazole and clarithromycin such as Iran. Our pilot study revealed that a very good *H. pylori* eradication rate could be achieved with a modified bismuth-based quadruple therapy containing high-dose amoxicillin (3 g/day), adequate-dose metronidazole (1.5 g/day), and a PPI. Therefore, this open-label, randomized clinical trial was designed to compare the classic bismuth-based quadruple regimen containing metronidazole (1500mg/day) with a modified bismuth-based quadruple therapy containing high-dose amoxicillin (3 g/day), metronidazole (1.5 g/day), and a PPI, with the aim to compare eradication rates, adverse effects, and patient compliance.

**Materials and Methods**

**Patient population**

This was a prospective, randomized, open-label clinical trial study conducted at Shahid Sadoughi University (SSU) of Medical Center, a tertiary care hospital located in Yazd, Iran. Criteria for inclusion in this study were as follows: no history of treatment for *H. pylori* eradication, age above 18 years, endoscopically confirmed diagnosis of duodenal ulcer, and positive rapid urease test. Patients with a history of previous gastric surgery, allergy to antibiotics, those who were treated with antibiotics in the preceding eight weeks, those with major systemic disease, and those who were pregnant or lactating were excluded from the study. The primary endpoint was *H. pylori* eradication rate by intention-to-treat (ITT) analysis. The secondary endpoint was frequency of adverse effects and treatment compliance.

**Intervention**

Patients were enrolled since October 20, 2014. After the patients provided consent to participate in this trial, they were randomly assigned at a 1:1 ratio to receive one of the following two treatment regimens. The amoxicillin group (group I) received metronidazole 500mg, amoxicillin 1000mg, bismuth subcitrate 240mg, all three times a day, plus omeprazole 20mg twice a day, for 14 days. The tetracycline group (group II) received omeprazole 20mg twice a day; bismuth 240 mg, and tetracycline HCl 500 mg, both four times a day; and metronidazole 500 mg three times a day, for 14 days. Omeprazole and bismuth were taken before meals, and antibiotics were used after meals. All patients were instructed on potential adverse effects and kept under observation during treatment for evaluation of adverse effects and compliance. All patients were requested to record any adverse effects that occurred during therapy, including bad taste, diarrhea, dizziness, weakness, nausea, loss of appetite, vomiting, fatigue, fever, and skin rash. Severe adverse effects were defined as those that would be considered to disrupt daily activities that required treatment discontinuation by the patient.

**Outcomes**

For evaluation for the primary outcome of *H. pylori* eradication rate, patients were asked to stop the PPI or the H2 blocker for at least four weeks before follow-up evaluation. Eight weeks after conclusion of the two-week study treatment, patients were assessed by the C13urease breath test by personnel who were blinded to the treatment, and a value of less than 4% was defined as successful *H. pylori* eradication. For evaluation of secondary outcomes, data on adverse effects were collected through a standard side effect questionnaire, and good compliance was defined as ingesting more than 80% of the total number of doses included in the regimen.

**Sampling and blinding**

Sample size for the trial was calculated based on the following assumptions. Average rate of successfully achieved eradication of *H. pylori* with the standard quadruple therapy was 80% (17). Based on our pilot study results showing an *H. pylori* eradication rate of 94% with the modified bismuth-based quadruple therapy containing high-dose amoxicillin (3 g/day), adequate-dose metronidazole dose (1.5 g/day) and omeprazole, we chose a two-sided alpha value of 0.05, and a power of 80%. Based on these assumptions at least 208 participate (104 subjects in each groups) would be required. In order to accommodate a 10% rate of lost to follow-up, we enrolled 228 patients.

**Statistical methods**

All registered data were analyzed using SPSS software version 22 for Windows (SPSS, Chicago, IL). Data were presented as means with standard deviation (SD), frequencies, and percentages. The chi-square and Fisher’s exact tests were used for comparison of categorical data between the two groups. *P* values of less than 0.05 were considered significant for all analyses. ITT and per-protocol analyses were performed to calculate eradication rates. The ITT analysis included all randomized patients. Individuals who did not take at least 80% of the drugs and those with unknown post-treatment *H. pylori* status were excluded from the PP analysis. Odds ratios with 95% confidence intervals (CIs) were calculated where appropriate.

**Ethical Consideration**

This study was approved by the Ethics Committee of Shahid Sadoughi University of Medical Sciences in Yazd, Iran and registered with the protocol number “Ir.ssu.rec.1394.13712” on September 20, 2014. Participants provided written informed consent and were included in the study after they were provided information on treatment methods. This trial was also registered with Thai Clinical Trial Registry (Number: TCTR20170623004)

Subject screening and treatment flowchart

Ambulatory patients who are receive *H. pylori* eradication regimen for the first time

Obtain informed consent

Enroll and randomize subjects in two groups:

1- Bismuth–based quadruple regimen containing high dose of amoxicillin

2 – Classic Bismuth–based quadruple therapy

Following drug treatment

For both quadruple therapy-14 days

First evaluation at Two week after starting of these therapies

Visit in ambulatory clinic and check

Compliance with treatment and reports of adverse effects

Compliance> 80%-

Satisfactory

Compliance < 80% -

Unsatisfactory

Second evaluation -8 weeks after completing treatment

C-urea breath test check the results of

Schedule of events

|  | **On enrollment** | **14 days after starting of treatment –visit in clinic** | **8 weeks after completing treatment** |
| --- | --- | --- | --- |
| Sign informed consent | X |  |  |
| Check inclusion/exclusion criteria | X |  |  |
| Start one of two treatment regimen  ( two quadruple therapy) | X |  |  |
| Check compliance |  | X |  |
| Ask about adverse effects |  | X |  |
| c-urea breath test |  |  | X |

**Enrollment:**

Ambulatory patients who have duodenal ulcer with positive H. pylori infection and receive H. pylori eradication regimen for the first time, will be approached by a member of the study research team. A study research staff member will explain the purpose, procedures and intent of the study to each potential participant. Interested subjects will be invited to join the study and asked to provide a written informed consent prior to initiation of any study-related procedure.

Subjects who failed to meet the clinical inclusion and exclusion criteria will not be included in the study. Eligible subjects will be randomized to one of the two treatment arms in a1:1 ratio Classic bismuth-based quadruple therapy and quadruple therapy, which tetracycline substitute with High dose of amoxicillin.

14 days after starting of treatment

First evaluation will take place two weeks after starting of the treatment. A study research staff member will ask each patient about the compliance and adverse effect.

8 weeks after completing treatment

Eight weeks after completing treatment the results of C13-urea breath will be checked by HELIC fan Germany.

**REFRENCESS:**

1. Go M. Natural history and epidemiology of Helicobacter pylori infection. Alimentary pharmacology & therapeutics. 2002;16(s1):3-15.

2. Khademi F, Poursina F, Hosseini E, Akbari M, Safaei HG. Helicobacter pylori in Iran: A systematic review on the antibiotic resistance. Iranian journal of basic medical sciences. 2015;18(1):2.

3. Fischbach L, Evans E. Meta‐analysis: the effect of antibiotic resistance status on the efficacy of triple and quadruple first‐line therapies for Helicobacter pylori. Alimentary pharmacology & therapeutics. 2007;26(3):343-57.

4. Lu H, Zhang W, Graham DY. Bismuth-containing quadruple therapy for Helicobacter pylori: lessons from China. European journal of gastroenterology & hepatology. 2013;25(10).

5. Graham DY, Fischbach L. Helicobacter pylori treatment in the era of increasing antibiotic resistance. Gut. 2010;59(8):1143-53.

6. Shokrzadeh L, Alebouyeh M, Mirzaei T, Farzi N, Zali MR. Prevalence of multiple drug-resistant Helicobacter pylori strains among patients with different gastric disorders in Iran. Microbial Drug Resistance. 2015;21(1):105-10.

7. Keshavarz Azizi Raftar S, Moniri R, Saffari M, Razavi Zadeh M, Arj A, Mousavi SGA, et al. The helicobacter pylori resistance rate to clarithromycin in Iran. Microbial Drug Resistance. 2015;21(1):69-73.

8. Farshad S, Alborzi A, Japoni A, Ranjbar R, Asl KH, Badiee P, et al. Antimicrobial susceptibility of Helicobacter pylori strains isolated from patients in Shiraz, Southern Iran. World Journal of Gastroenterology: WJG. 2010;16(45):5746.

9. Graham DY, Lee YC, Wu MS. Rational Helicobacter pylori therapy: evidence-based medicine rather than medicine-based evidence. Clinical Gastroenterology and Hepatology. 2014;12(2):177-86. e3.

10. Fischbach L, Zanten S, Dickason J. Meta‐analysis: the efficacy, adverse events, and adherence related to first‐line anti‐Helicobacter pylori quadruple therapies. Alimentary pharmacology & therapeutics. 2004;20(10):1071-82.

11. Calvet X, Ducons J, Guardiola J, Tito L, Andreu V, Bory F, et al. One‐week triple vs. quadruple therapy for Helicobacter pylori infection—a randomized trial. Alimentary pharmacology & therapeutics. 2002;16(7):1261-7.

12. Goodwin C, Marshall B, Blincow E, Wilson D, Blackbourn S, Phillips M. Prevention of nitroimidazole resistance in Campylobacter pylori by coadministration of colloidal bismuth subcitrate: clinical and in vitro studies. Journal of clinical pathology. 1988;41(2):207-10.

13. Laine L, Hunt R, El-Zimaity H, Nguyen B, Osato M, Spénard J. Bismuth-based quadruple therapy using a single capsule of bismuth biskalcitrate, metronidazole, and tetracycline given with omeprazole versus omeprazole, amoxicillin, and clarithromycin for eradication of Helicobacter pylori in duodenal ulcer patients: a prospective, randomized, multicenter, North American trial. The American journal of gastroenterology. 2003;98(3):562-7.

14. Abadi AT, Taghvaei T, Mobarez AM, Carpenter BM, Merrell DS. Frequency of antibiotic resistance in Helicobacter pylori strains isolated from the northern population of Iran. Journal of microbiology (Seoul, Korea). 2011;49(6):987-93.

15. Kim JJ, Reddy R, Lee M, Kim JG, El-Zaatari FA, Osato MS, et al. Analysis of metronidazole, clarithromycin and tetracycline resistance of Helicobacter pylori isolates from Korea. The Journal of antimicrobial chemotherapy. 2001;47(4):459-61.

16. Mendonca S, Ecclissato C, Sartori MS, Godoy AP, Guerzoni RA, Degger M, et al. Prevalence of Helicobacter pylori resistance to metronidazole, clarithromycin, amoxicillin, tetracycline, and furazolidone in Brazil. Helicobacter. 2000;5(2):79-83

17. A Fischbach L, Evans EL Meta-analysis: the effect of antibiotic resistance status on the efficacy of triple and quadruple first-line therapies for Helicobacter pylori. Aliment Pharmacol Ther. 2007 Aug 1; 26(3):343-57.
