# Supplementary material for: Efficacy and Tolerability of Two Quadruple Regimens: Bismuth, Omeprazole, Metronidazole with Amoxicillin or Tetracycline as First-Line Treatment for Eradication of Helicobacter Pylori in Patients with Duodenal Ulcer: A Randomized Clinical Trial
Source: PLoS One. 2018 Jun 11;13(6):e0197096. doi: 10.1371/journal.pone.0197096 (PMC5995362; doi:10.1371/journal.pone.0197096)
Supplement: S2 File — (PDF) [file pone.0197096.s003.pdf]

**Manuscript Type:** Clinical Trial Study

**Title:** Efficacy and Tolerability of Two Quadruple regimens: Bismuth, Omeprazole, Metronidazole and Amoxicillin or Tetracycline as First-line Treatment for eradication of Helicobacter pylori in patients with duodenal ulcer

**Correspondence to: Hassan Salman Roghani , MD,** Panceratobiliary disease Research center, Division of gastroenterology, Department of Internal Medicine, Shahid sadoughi University of Medical Sciences, Yazd, Iran .

**Email:** H.salmanroghani@ssu.ac.ir

**Telephone:** +98-353-8224000

**Fax:** +98-353-7254750

The study was reviewed and approved by the institute ethics committee at shahid Sadoughi University of Medical Sciences, Yazd, Iran, Ethic Number "Ir.ssu.rec.1394.13712"
